# The double-edged sword effects of active social media use on loneliness: The roles of interpersonal satisfaction and fear of missing out

**DOI:** 10.3389/fpsyg.2023.1108467

**Published:** 2023-02-09

**Authors:** Jian Mao, Ge-xi Fu, Jian-jun Huang

**Affiliations:** ^1^The School of Humanities, Jiangxi University of Chinese Medicine, Nanchang, China; ^2^Key Laboratory of Psychology of TCM and Brain Science, Jiangxi Administration of Traditional Chinese Medicine, Jiangxi University of Chinese Medicine, Nanchang, China

**Keywords:** loneliness, active social media use, interpersonal relationship satisfaction, fear of missing out, online-specific state-FoMO, general trait-FoMO

## Abstract

**Introduction:**

Social media' impact on loneliness has attracted widespread scholarly attention. One hypothesis is that active social media use (ASMU) is associated with a decrease in loneliness. However, several empirical studies did not find a significant correlation between ASMU and loneliness, and ASMU may even increase loneliness. This study explored the mechanism of the double-edged sword effects of ASMU on loneliness.

**Methods:**

Data were collected through convenience sampling from three universities in China. A total of 454 Chinese college social media users (Mean age 19.75 ± 1.33; 59.92% female) completed an online questionnaire.

**Results:**

ASMU was positively related to interpersonal relationship satisfaction, which was negatively related to general trait-fear of missing out (FoMO) and loneliness. Further structural equation modeling (SEM) analysis showed that ASMU could negatively predict loneliness through the mediation pathways of interpersonal satisfaction and “Interpersonal satisfaction → Trait-FoMO.” At the same time, ASMU was also positively associated with online-specific state-FoMO, which was positively associated with trait-FoMO and loneliness. Further SEM analysis found no mediation effect of state-FoMO between ASMU and loneliness, but state-FoMO and traitFoMO sequentially mediate the relationship between ASMU and loneliness.

**Discussion:**

This study indicates that ASMU may increase and decrease loneliness. Interpersonal satisfaction and FoMO explained the double-edged mechanism of ASMU on loneliness. These findings contribute to dialectically understanding the effectiveness of active social media use and provide theoretical guidance for promoting the beneficial aspects of social media while weakening its harmful consequences.

## 1. Introduction

Over the past decade, the impact of social media on individuals' mental health and wellbeing has been conducted by researchers in many disciplines (e.g., psychology, communication, information science, and clinical medicine) (Wang et al., 2018; Kross et al., 2021; Valkenburg, 2021). As research progressed, researchers have consciously categorized and integrated social media usage behaviors, classifying two main types: active social media use (ASMU) and passive social media use (PSMU) (Verduyn et al., 2017). ASMU refers to users' behavior of communicating directly with others on social media, such as proactively sending messages to friends and updating their status (Verduyn et al., 2017). PSMU mainly refers to information-browsing behaviors that lack communication, such as browsing other people's updates (Verduyn et al., 2017). Different types of usage uniquely impact an individual's self and psychosocial adjustment. Empirical studies have shown that ASMU is associated with positive effects, such as enhanced self-esteem (Marengo et al., 2021), improved friendship quality (Valkenburg and Peter, 2009; Trepte et al., 2018), increased social support (Choi, 2022), and increased life satisfaction (Choi, 2022). PSMU reduces positive emotions (Sun et al., 2022), promotes harmful social comparisons and stress (Yue et al., 2022), diminishes self-esteem (Cheng and Nhan, 2021), and reinforces loneliness (Zhang L. et al., 2022).

Loneliness is a common phenomenon in modern society and is considered a global public health issue (Cacioppo and Cacioppo, 2018). Loneliness describes a subjective negative emotional experience related to social communication. The discrepancy between individuals' desired and existing social relationships causes feelings of loneliness (Peplau and Perlman, 1982). Loneliness impairs individuals' wellbeing (Stieger et al., 2021) and leads to more emotional (Van As et al., 2021) and behavioral problems (Zhang and Xiang, 2022), and suicidal tendencies happen in severe cases (Gijzen et al., 2021). One hypothesis that garnered much attention is that ASMU behaviors such as direct communication and self-disclosure can reduce loneliness because it promotes positive social connections and social support (Lee et al., 2013; Verduyn et al., 2017). An experimental study found that online posting behaviors reduced individuals' loneliness by increasing social connectivity (Deters and Mehl, 2013). Other empirical studies have also shown that ASMU can reduce loneliness (Brown et al., 2021; Yang et al., 2021; Lin et al., 2022; Zhang L. et al., 2022).

However, recent studies have reached inconsistent conclusions. Several empirical studies found no significant direct association between ASMU and loneliness (Dibb and Foster, 2021; Quynh Ho and Nguyen, 2022). Two meta-analysis studies found that loneliness was significantly and positively related to PSMU, but loneliness was not significantly related to ASMU (Yin et al., 2019; Zhang L. et al., 2022). Some scholars have argued that ASMU and loneliness are in an inverted U-shaped relationship. Their longitudinal study suggests that only appropriate ASMU reduces loneliness and that less or too much use is detrimental to alleviating loneliness (Wang et al., 2018). Scholars who dispute the active use hypothesis have pointed out that the association between ASMU and psychological indicators related to wellbeing or ill-being is much more complex than a direct relationship between the two (Beyens et al., 2020, 2021). In other words, rather than answering whether ASMU increases or decreases wellbeing, researchers should explore what mechanisms ASMU influences individuals' wellbeing. Who are susceptible? Under what conditions are they susceptible? (Kross et al., 2021; Valkenburg, 2021; Valkenburg et al., 2022). The above perspectives indicate that ASMU may increase and decrease loneliness. Moreover, the different impacts can be explained by different underlying mechanisms behind the relationships. Most previous studies have explored how ASMU lowers loneliness, not how it may increase it.

Furthermore, examining the effectiveness of ASMU only from a one-sided perspective (positive or negative) is unilateral. This limitation must be broken, and a comprehensive and discriminatory view of ASMU must be taken to explore its potential double-edged effects on loneliness, which is the focus of this study. Below, we will specifically explore these impact pathways.

### 1.1. Positive effects: The mediating role of interpersonal relationship satisfaction

How ASMU is associated with a reduction in loneliness? Interpersonal relationship satisfaction can explain that. ASMU may be associated with increased interpersonal relationship satisfaction as positive social behavior. Communication and interaction are prerequisites for good interpersonal relationships (Altman and Taylor, 1973). ASMU can maintain and enhance users' social connections (Van Ouytsel et al., 2016; Iannone et al., 2018), promote mutual acquaintance among friends, and thus improve the sense of connection and friendship quality between the parties involved (Utz, 2015; Wang et al., 2017). Longitudinal studies have confirmed the positive predictive effect of ASMU on interpersonal relationship quality (Valkenburg and Peter, 2009; Trepte et al., 2018). The cognitive processing theory suggests that loneliness arises when individuals are more dissatisfied with their interpersonal relationships than expected (Peplau and Perlman, 1982). Tian et al. (2019) demonstrated that interpersonal satisfaction negatively predicted the level of loneliness 8 months later.

Thus, we hypothesized that interpersonal relationship satisfaction mediates the relationship between ASMU and loneliness; that is, interpersonal satisfaction will explain the association between ASMU and decreased loneliness (H1).

### 1.2. Negative effects: The mediating role of state-FoMO

Previous research has demonstrated that fear of missing out plays a vital mediator linking social media use to numerous adverse outcomes (Kuss and Griffiths, 2017; Tandon et al., 2021). FoMO was originally defined as generalized anxiety caused by the fear of missing out on rewarding experiences shared by others (Przybylski et al., 2013). Recently, FoMO was considered a two-dimensional construct comprising general trait-FoMO and online-specific state-FoMO (Wegmann et al., 2017). The definition of trait-FoMO is consistent with Przybylski et al. (2013). State-FoMO refers to the concerns about missing information online. It is an unstable cognitive bias apparent during certain online activities, including social media use (Wegmann et al., 2017; Röttinger et al., 2021). That is, state-FoMO is directly caused by frequent internet usage (Wegmann et al., 2017).

As social media behavior, ASMU may trigger state-FoMO. Media effects theories state that media use behavior may reinforce certain cognitive, emotional, and behavioral aspects of users (Valkenburg et al., 2016), especially media-related perceptions (Kim, 2009). Thus, ASMU positively affects interpersonal maintenance but may trigger or reinforce state-FoMO in this process because of the properties of social media (Valkenburg et al., 2016). First, unlike offline socializing, online socializing can be initiated at any time (Zhou and Liu, 2016), dramatically increasing the possibility of connecting and sharing with acquaintances and the concern of missing out on online information (Wegmann et al., 2017). Another difference from real-life social interaction is the asynchronous nature of online social interaction (Zhou and Liu, 2016). When users update or send messages to their friends, they only sometimes receive immediate replies, and they may mostly have to wait for others to reply or like and comment (Alutaybi et al., 2019a), prompting more social media checking behavior among users (Wegmann et al., 2017). Thus, assuming that frequent active social media use may have increased state-FoMO is reasonable.

Furthermore, state-FoMO is a proximal factor in developing problematic social media use behaviors (Wegmann et al., 2017; Röttinger et al., 2021). A study showed that problematic social media use positively predicted loneliness 15 months later (Marttila et al., 2021). Chen et al. (2022) study found that problematic social media use increased depressive symptoms and that loneliness mediates this relationship. Thus, state-FoMO may be associated with increased loneliness. Therefore, we hypothesized that state-FoMO mediates the relationship between ASMU and loneliness; that is, state-FoMO will explain the association between ASMU and increased loneliness (H2).

### 1.3. The sequential mediation of the double-edged sword effects: The role of trait-FoMO

Interpersonal satisfaction and state-FoMO may indirectly predict loneliness *via* trait-FoMO. Despite the growing literature on FoMO (Tandon et al., 2021), few studies have distinguished between trait- and state-FoMO. Conceptually, trait-FoMO is a cognitive tendency and exhibits the apparent state of jealousy and absence of psychological need (Röttinger et al., 2021; Yin et al., 2021). In contrast, state-FoMO is not as problematic as trait-FoMO, which represents more unstable cognition that accompanies social media use (Wegmann et al., 2017; Röttinger et al., 2021). Recent investigations reveal that trait anxiety and depression are significantly related to trait-FoMO but not state-FoMO (Wegmann et al., 2017; Balta et al., 2020). Moreover, studies have found a stronger association between state-FoMO and social media use intensity than trait-FoMO (Wegmann et al., 2017; Li et al., 2020; Röttinger et al., 2021), suggesting that social media use increasing FoMO should follow the process: “Social media use to State-FoMO to Trait-FoMO.”

In addition, although trait-FoMO is a relatively stable cognitive tendency, similar to other relatively stable variables (e.g., self-esteem), it possesses some stability while being influenced by interpersonal and situational factors (Przybylski et al., 2013; Chai et al., 2018). Trait-FoMO has been interpreted as a result of unmet basic psychological needs (especially need to belong) (Przybylski et al., 2013; Lai et al., 2016). The need to belong theory states that individuals feeling satisfied with interpersonal relationships means belonging is satisfied (Baumeister and Leary, 1995). With this, it is inferred that higher interpersonal relationship satisfaction will reduce concern for others and experience less trait-FoMO. Therefore, higher interpersonal satisfaction may be associated with lower trait-FoMO.

Self-determination theory is the most commonly used theory to explain trait-FoMO (Tandon et al., 2021). Researchers have linked trait-FoMO to unmet basic psychological needs (Przybylski et al., 2013; Dou et al., 2021). According to self-determination theory, unmet basic psychological needs can lead to various negative consequences, including feelings of loneliness (Ryan and Deci, 2000; Saricali and Guler, 2022). Thus, trait-FoMO may lead to an increase in loneliness. Prior cross-sectional research has demonstrated a positive association between the trait-FoMO and loneliness (Fumagalli et al., 2021; Alinejad et al., 2022). Moreover, studies with Experience Sampling Method (ESM) have shown that daily trait-FoMO increases individuals' negative affect, such as anxiety, depression, and stress, the next day and decreases positive affect (Milyavskaya et al., 2018; Elhai et al., 2020).

Based on the above analysis, we hypothesized that interpersonal relationship satisfaction and trait-FoMO will sequentially mediate the relationship between ASMU and loneliness (H3), and state-FoMO and trait-FoMO will sequentially mediate the relationship between ASMU and loneliness (H4).

### 1.4. Present study

**Figure 1:**
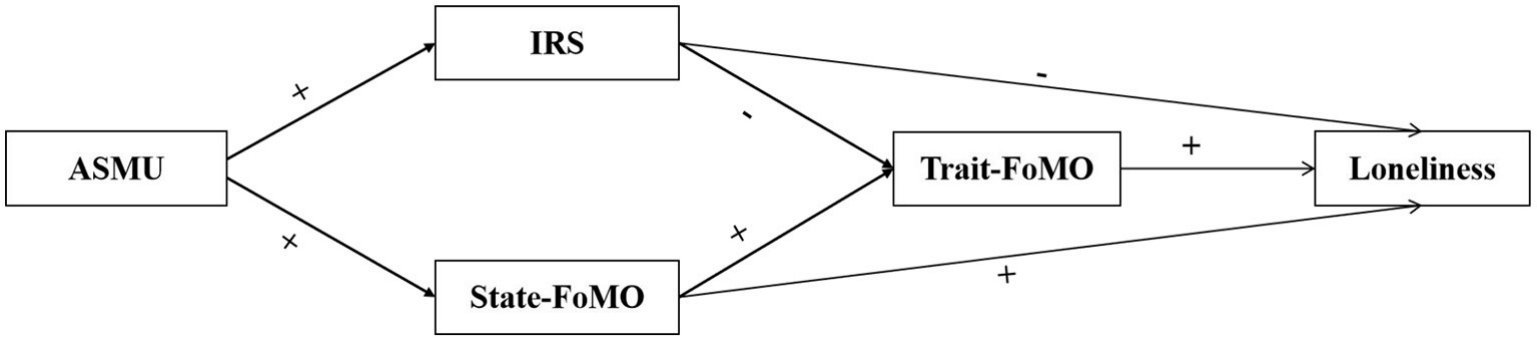
Proposed research model. Note: ASMU: Active Social Media Use; IRS: Interpersonal Relationship Satisfation; FoMO: Fear of Missing Out.

This study tested a complex mediation model (see Figure 1) to shed light on the potential mechanism of the double-edged sword effect of ASMU on loneliness. Positive effects mechanisms explored how ASMU decreased loneliness, including (1) the mediating role of interpersonal satisfaction and (2) the sequential mediating roles of interpersonal satisfaction and trait-FoMO. Harmful effects mechanisms explored how ASMU increased loneliness, including (1) the mediating role of state-FoMO and (2) the sequential mediation roles of state-FoMO and trait-FoMO. It is important to note that ASMU and PSMU are not two separate activities but exist in co-occurrence (Verduyn et al., 2017). Moreover, Longitudinal studies have shown that PSMU is essential in increasing the trait-FoMO and state-FoMO (Ma, 2020; Zhang et al., 2021). PSMU was also negatively associated with relationship quality and positively associated with loneliness (Zhang F. J. et al., 2022; Zhang L. et al., 2022). Therefore, the effect of PSMU needs to be excluded to increase the scientific accuracy of the effect size of ASMU affecting the double-edged pathway of loneliness. Referring to the approach in the study by Yin et al. (2022), we measured the PSMU intensity of individuals and controlled for it in a hypothetical model test.

## 2. Materials and methods

### 2.1. Participants

This study primarily focused on participants' active use behaviors such as chatting, posting, and commenting on three social media platforms, QQ, WeChat, and Sina Weibo.QQ and WeChat are mainly based on acquaintance social networks. QQ and WeChat are not only instant messaging APPs but also include a social networking site for Qzone and WeChat's Moments, respectively. Sina Weibo is a public platform for instant information sharing and communication. The survey was conducted in May–June 2021. Convenience sampling method was used to select 537 college students from three universities in Jiangxi province as respondents. A total of 490 undergraduate students with experience (more than 1 year) in social media use voluntarily completed the survey. Of those who completed the survey, 9 students reported in the last item of the questionnaire that the questionnaire was not filled out carefully, 21 answered too short (< 2 min), and 6 had incomplete demographic information. After excluding these questionnaires, 454 valid questionnaires were obtained from 182 males (40.08%) and 272 females (59.92%) respondents, ranging from 18 to 27 years (M = 19.75; SD = 1.33). Among the qualified respondents, 105 are first-year students, 225 sophomores, 85 juniors, and 38 seniors. The average time spent using social media is 3.62 h per day.

### 2.2. Procedure

This survey was conducted online (a Chinese survey website: www.wjx.cn). We contacted teachers at three universities in Jiangxi province and asked them to send the online questionnaire link to the class QQ notification group of the classes they teach. The classes are 10 in total. These students were told that they would take a psychological test and that it was a course assignment they needed to complete as carefully as possible. All students who participated in the survey understood the purpose and were aware of the principle of confidentiality and voluntariness. They all checked the “Consent” box in the informed consent section on the first page of the questionnaire. This study involving humans was conducted following the ethical standards of the Declaration of Helsinki and with the ethical regulations of the first author and the corresponding author's university.

### 2.3. Measures

#### 2.3.1. Active social media use

The ASMU subscale of the “Surveillance Use” scale (Tandoc et al., 2015), revised by Liu et al. (2017), was used. The scale consists of four questions (e.g., “proactive messaging of friends”), and each is scored out of a 5-point scale (1 = never, 5 = frequently). A higher score indicates a high ASMU frequency. Our samples' Cronbach's alpha was 0.72, 0.70, for McDonald's omega, which indicates good reliability. The scale has acceptable convergent validity because the average variance extracted (AVE) was 0.43. Good fit indicators for the confirmatory factor analysis (CFA): χ2/*df* = 3.484, CFI = 0.960, TLI = 0.944, SRMR = 0.057, RMSEA = 0.074, indicates the construct validity is good.

#### 2.3.2. Trait-state fear of missing out

Wegmann et al. (2017) developed the Trait-State Fear of Missing Out Scale, which was used in this study. Li et al. (2020) showed that the Chinese version of the scale has good reliability and validity in Chinese culture. The scale consists of 12 questions, five on trait-FoMO (e.g., “I fear my friends have more rewarding experiences than me”) and seven on state-FoMO (e.g., “I continuously consult my smartphone in order not to miss out on (anything)”). A 5-point Likert scale (1 = totally disagree, 5 = totally agree) was used, with higher scores indicating higher levels of trait- or state-FoMO. Our sample's Cronbach's alpha of the trait-FoMO subscale was 0.82, 0.81 for McDonald's omega, which indicates good reliability. The AVE was 0.50. Good fit indicators for the CFA: χ2/*df* = 5.569, CFI =0.986, TLI = 0.955, SRMR = 0.037, RMSEA = 0.100, indicates the construct validity is good. Cronbach's alpha of the state-FoMO subscale was 0.85, 0.84 for McDonald's omega, which indicates good reliability. The AVE was 0.45. Good fit indicators for the CFA: χ2/*df* = 4.310, CFI = 0.970, TLI = 0.948, SRMR = 0.035, RMSEA = 0.085, indicates the construct validity is good.

#### 2.3.3. Loneliness

The UCLA loneliness scale developed by Russell (1996) was used to evaluate participants' levels of loneliness. Guo et al. (2017) showed that the Chinese version of the scale has good reliability and validity in Chinese culture. The scale consists of 20 questions, 11 positive scoring questions (e.g., “Do you often feel lonely”), and 9 reverse-scored questions (e.g., “Do you often feel close to people”). The response is scored on a four-point Likert scale (1 = not at all, 4 = fully). After the reverse scoring of the reverse questions, individuals were more lonely when they got higher scores. Our sample's Cronbach's alpha was 0.87, 0.87 for McDonald's omega, indicates good reliability. The AVE was 0.43. The fit indicators for the CFA: χ2/*df* = 3.484, CFI =0.903, TLI = 0.890, SRMR = 0.070, RMSEA = 0.069, indicates an acceptable construct validity.

#### 2.3.4. Interpersonal relationship satisfaction

Referring to Mellor et al. (2008), a single item measures interpersonal relationship satisfaction. This item asks “How satisfied are you with your interpersonal relationships?” with a 7-point scale (1 = very dissatisfied, 7 = very satisfied).

#### 2.3.5. Control variables

The PSMU subscale of the “Surveillance Use” scale (Tandoc et al., 2015), revised by Liu et al. (2017), was used. The scale consists of four questions (e.g., “View photos uploaded by others”) with a 5-point scale (1 = never, 5 frequently); and higher scores indicate higher degrees of PSMU. Our samples' Cronbach's alpha and McDonald's omega were 0.83 and 0.84, respectively, which indicates good reliability. The AVE was 0.56. The fit indicators for the confirmatory factor testing: χ2/*df* = 3.430, CFI = 0.997, TLI = 0.981, SRMR = 0.015, RMSEA = 0.076, indicates an acceptable construct validity. Previous research has shown that time spent on social media use is positively associated with trait-FoMO and state-FoMO (Fioravanti et al., 2021). Thus, this study also collected the participants' per day social media use time as a control variable. Participants were asked to report the average daily time (hours) spent using social media over the last week.

### 2.4. Data analysis

The study was statistically analyzed using SPSS 22.0 and AMOS 23.0. Using SPSS 22.0 to store and manage the data, check the internal consistency reliability of instruments, test for common method bias, perform descriptive statistical analysis and calculate Pearson correlations between variables. The convergent validity and structural validity of the instruments were checked using AMOS 23.0. We then tested the structural equation modeling (SEM) in the study hypothesis using AMOS 23.0. The steps included the following: (1) Item parceling. The present study had many measurement items for individual variables (e.g., loneliness). The large number of parameters to be estimated in the model may increase the standard errors (Little et al., 2002). Thus, in this study, all four latent variables (ASMU, Trait-FoMO, State-FoMO, and Loneliness) were parceled (Item parceling). According to Rogers and Schmitt's (2004) suggestion, the measurement items of each latent variable were parceled into three indicators using the factorial algorithm parceling method. (2) Constructing and testing the structural equation modeling. Together with two control variables (both observed), the structural model includes four latent and 15 observed variables. (3) Estimating confidence intervals for mediating effects using the bias-corrected non-parametric percentile bootstrap method, with a 95% CI not containing 0, indicating statistical significance (Wen and Ye, 2014).

Cronbach's α and McDonald's Omega coefficients were used to assess reliability, with >0.70 indicating good reliability (McDonald, 2013). Convergent validity was assessed using AVE, with >0.36 indicating an acceptable and >0.5 an ideal convergent validity (Chin, 1998). Using the maximum likelihood (ML) method to process the models. This study judged the model fit by the magnitude of the following indices: Tucker Lewis Index (TLI), standardized root mean square residual (SRMR), comparative fit index (CFI), and root-mean-square error of approximation (RMSEA). According to the recommendation (Kenny, 2015), CFI, TLI > 0.90 is considered acceptable, and > 0.95 is considered a good fit. SRMR < 0.08 is considered a good fit, < 0.08 for RMSEA. However, we rely more on SRMR than RMSEA because RMSEA is often more likely to misjudge a poor fit than SRMR (Shi et al., 2020).

## 3. Results

### 3.1. Common method biases and multicollinearity testing

Given the self-reported nature of the data, we are employed the Harman single-factor method checking for common method bias before statistical analysis. The results revealed that the first factor explained 29.3% (< 40%) of the total variance in the data, indicating no serious problem of common method bias in this study (Podsakoff et al., 2012). We tested the variables for possible multicollinearity problems using the variance inflation factor (VIF) diagnostic method. The results revealed that the VIF values of the respective variables in this study were < 2, indicating no multicollinearity problem (Hair et al., 2019).

### 3.2. Preliminary analyses

Table 1 shows all observed variables' means, standard deviations, and the correlation matrix. ASMU was positively correlated with state-FoMO, trait-FoMO, and interpersonal satisfaction; ASMU was negatively correlated with loneliness. State-FoMO and trait-FoMO were positively correlated with loneliness. Interpersonal satisfaction was negatively correlated with trait-FoMO and loneliness. PSMU and daily SM use time were positively correlated with trait-FoMO and state-FoMO.

**Table 1:** Descriptive statistics and interrelations among observed variables.

	**1**	**2**	**3**	**4**	**5**	**6**	**7**	**8**	**9**
1 ASMU	1								
2 State-FoMO	0.342^***^	1							
3 IRS	0.202^***^	0.004	1						
4 Trait-FoMO	0.155^*^	0.609^***^	−0.186^***^	1					
5 Lonelines	−0.169^***^	0.129^**^	−0.384^***^	0.345^***^	1				
6 PSMU	0.469^***^	0.339^***^	0.038	0.232^***^	0.026	1			
7 Gender	0.107^*^	−0.024	−0.010	0.048	0.047	0.064	1		
8 Age	−0.066	−0.014	−0.050	−0.024	0.044	−0.020	−0.063	1	
9 Daily SMU Time	0.205^***^	0.145^**^	−0.009	0.134^**^	−0.023	0.200^***^	0.100^*^	−0.047	1
*M*	2.57	2.66	4.85	2.62	2.23	3.01		19.75	3.62
*SD*	0.64	0.72	1.42	0.74	0.38	0.77		1.33	2.63

*N* = 454. ^*^ *p* < 0.05, ^**^ *p* < 0.01, ^***^ *p* < 0.001; male = “1,” female = “2”. ASMU:Active Social Media Use; PSMU: Passive Social Media Use; IRS: Interpersonal Relationship Satisfation. FoMO: Fear of Missing Out. Daily SMU Time:daily social media use time (hours).

### 3.3. Structural model testing

**Figure 2:**
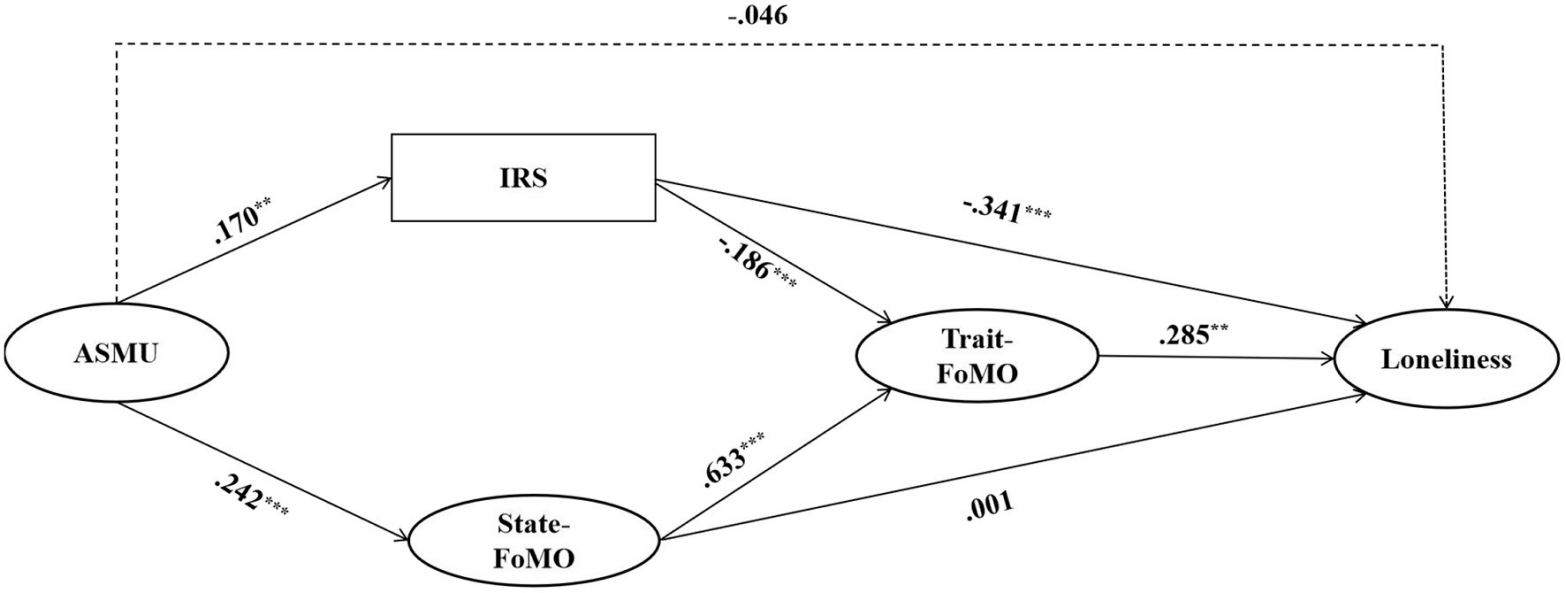
Final standardized parameter values of the mode. *N* = 454, ^**^p < 0.01, ^***^p < 0.001. For brevity, the paths of the control variables are not shown.

Controlling for PSMU and daily SM use time, the SEM results showed that the model fit index met acceptable criteria: (χ^2^/*df* = 4.123, CFI = 0.926, TLI = 0.900, SRMR = 0.066, RMSEA = 0.083). The detailed standardized path coefficients are shown in Figure 2. The direct effect of ASMU on loneliness was not significant (β = −0.046, *p* > 0.05, 95% CI [−0.162, 0.058]). ASMU positively predicted interpersonal satisfaction (β = 0.170, *p* < 0.01, 95% CI [0.077, 0.260]), interpersonal satisfaction negatively predicted loneliness (β = −0.341, *p* < 0.001, 95% CI [−0.415, −0.261]). ASMU positively predicted state-FoMO (β = 0.242, *p* < 0.001, 95% CI [0.139, 0.339]), and state-FoMO did not significantly predict loneliness (β = −0.001, *p* > 0.05, 95% CI [−0.135, 0.154]). It means that hypothesis 2 wil not be supported. Interpersonal satisfaction negatively predicted trait-FoMO (β = −0.186, *p* < 0.001, 95% CI [−0.262, −0.112]); State-FoMO positively predicted trait-FoMO (β = 0.633, *p* < 0.001, 95% CI [0.547, 0.715]), and trait-FoMO positively predicted loneliness (β = 0.285, *p* < 0.01, 95% CI [0.140, 0.416]).

Hypothesis 2 was not held, and we used the Bias-Corrected Bootstrap procedure to test for mediating effects in hypotheses 1, 3, and 4. Table 2 shows the results, and the 95% confidence interval of the mediating effect of each path does not contain 0, indicating that each mediating path is valid. Therefore, hypotheses 1, 3, and 4 were verified.

**Table 2:** Standardized indirect effects and their 95% confidence intervals.

**Indirect pathway**	**Indirect effect**	***SE***	**95% CI**	
			**Lower**	**Upper**
ASMU**→**IRS**→**Loneliness	−0.057	0.021	−0.093	−0.025
ASMU**→**IRS**→**Trait–FoMO**→**Loneliness	−0.009	0.005	−0.018	−0.003
ASMU**→**State–FoMO**→**Trait-FoMO**→**Loneliness	0.048	0.018	0.017	0.076

## 4. Discussion

The present study explored the mechanisms of the positive and negative effects of ASMU on loneliness. As shown in the correlation table, correlations for all main variables were significant except for PSMU, which was not significantly associated with interpersonal satisfaction and loneliness. On the one hand, the effect size of PSMU associated with relationship quality in the previous study was small (*r* = −0.08) (Zhang F. J. et al., 2022). On the other hand, the previous study measured individuals' assessment of the quality of their close friends' friendships. In contrast, the present study measured individuals' feelings about their interpersonal satisfaction, which PSMU may not influence. Surprisingly, PSMU was not significantly correlated with loneliness. However, several studies have found that PSMU also increases feelings of social connectedness and social presence (Orben et al., 2018; Yang et al., 2021), which may contribute to the overall non-significance of PSMU with loneliness. Further research is needed to explore the relationship between PSMU and loneliness. The present study used PSMU as a control variable to examine the double-edged mechanism by which ASMU affects loneliness.

The results supported hypotheses 1, 3, and 4, and did not support hypothesis 2. First, ASMU was positively related to interpersonal satisfaction, which was negatively related to trait-FoMO and loneliness. Further SEM analysis showed that ASMU could negatively predict loneliness through the mediation pathways of interpersonal satisfaction and “Interpersonal satisfaction → Trait-FoMO.” At the same time, ASMU was also positively associated with state-FoMO, which was positively associated with trait-FoMO and loneliness. Further SEM analysis found no mediation effect of state-FoMO between ASMU and loneliness, but state-FoMO and trait-FoMO sequentially mediate the relationship between ASMU and loneliness. These findings contribute to understanding how ASMU affects feelings of loneliness and its internal mechanisms and provide some insight into relevant practical applications.

### 4.1. Positive and negative mechanisms of active social media use on loneliness

These findings showed that interpersonal satisfaction mediated the link between ASMU and loneliness. More specifically, ASMU was associated with higher levels of interpersonal satisfaction, resulting in lower levels of loneliness. This result is consistent with the views that ASMU has positive psychological effects (Verduyn et al., 2017; Marengo et al., 2021; Choi, 2022). ASMU is a positive social behavior because it promotes communication and connection between people and increases social connection (Deters and Mehl, 2013; Utz, 2015; Brown et al., 2021). Furthermore, ASMU also enhances friendship quality by increasing the frequency of those who receive positive feedback from their friends (Lian et al., 2017). These could explain the positive effect of ASMU on increasing interpersonal relationship satisfaction. In turn, good interpersonal relationships can protect against loneliness (Tian et al., 2019).

Moreover, interpersonal satisfaction and trait-FoMO sequentially mediated the relationship between ASMU and loneliness, thus constituting the path of “ASMU → Interpersonal satisfaction → Trait-FoMO → Loneliness.” Specifically, interpersonal satisfaction reinforced by ASMU not only directly predicted loneliness but also predicted loneliness *via* the indirect effect of trait-FoMO. Satisfying with relationships indicates meeting social needs and having positive emotions (Baumeister and Leary, 1995; Verhagen et al., 2018). Therefore, individuals more satisfied with their relationships are less likely to perceive others as having more rewarding experiences and are less likely to be jealous of their friends (Yin et al., 2021), thus experiencing less trait-FoMO. Consistent with previous research, trait-FoMO is positively associated with loneliness (Fumagalli et al., 2021; Alinejad et al., 2022). Trait-FoMO results from the absence of social needs (Przybylski et al., 2013; Wegmann et al., 2017). Moreover, individuals with high trait-FoMO often believe that others are better off than they are (Röttinger et al., 2021; Servidio et al., 2021), have a sense of being abandoned by their friends (Fumagalli et al., 2021), and thus tend to feel lonely. Of course, individuals who feel lonely may also experience more trait-FoMO (Alinejad et al., 2022). It is necessary to conduct longitudinal studies to examine the possible bi-directional relationship between trait-FoMO and loneliness.

In contrast to our hypothesis, we did not find that state-FoMO mediated the relationship between ASMU and loneliness. The SEM results showed that state-FoMO did not predict loneliness, but trait-FoMO positively predicted loneliness, suggesting that trait-FoMO rather than state-FoMO increased the risk of loneliness. The results support the view of Wegmann et al. (2017) that FoMO is indeed a complex construct and that trait-FoMO is more problematic than state-FoMO (Röttinger et al., 2021). The results of the present study also showed that interpersonal relationship satisfaction was associated with low trait-FoMO but not significantly associated with state-FoMO. These results provide empirical evidence for differences between the two types of FoMO. Such evidence was not the present study's focus, but more research into the distinctions between trait-FoMO and state-FoMO is required.

We found a sequential mediating role of state-FoMO and trait-FoMO between ASMU and loneliness. State-FoMO reinforced by ASMU could positively predict loneliness *via* trait-FoMO. The finding provides a possible explanation for previous findings that ASMU increases loneliness (e.g., Wang et al., 2018), suggesting that state-FoMO plays an important role. The finding that ASMU was related to high state-FoMO echoes the theoretical claim of media use (Valkenburg et al., 2016) that media use behavior influences technology-related perceptions (Kim, 2009). Communication on social media is multiple and can occur at any time (Zhou and Liu, 2016), leading to users getting more information from many interactions and increasing the perception of missing information (Wegmann et al., 2017; Alutaybi et al., 2019a). Communication on social media is also asynchronous. Whether we initiate a session or post a message, receiving a response on time cannot be guaranteed (Zhou and Liu, 2016). This uncertainty makes individuals check social media more frequently to prevent missing messages (Alutaybi et al., 2019b). As Alutaybi et al. (2019a) described, “*Sending a message to someone for some purpose and waiting for a spontaneous response from the recipient can make people preoccupied.”* Thus, as the intensity of ASMU increases, users experience more state-FoMO, and frequent state-FoMO further stimulates trait-FoMO, thereby increasing the risk of experiencing loneliness.

Therefore, the association of ASMU with increased state-FoMO is an essential finding of this study because the relationship has not been directly tested in previous studies. Besides explaining the increase in loneliness, the increase in state-FoMO may also explain the findings that ASMU is negatively associated with wellbeing (Beyens et al., 2021) and positively associated with depression (Thorisdottir et al., 2019), which needs to be explored further in future empirical studies.

### 4.2. Theoretical and practical implications

Confronted with the previous inconsistent results on the association between active use and loneliness, we did not argue unilaterally that active use is associated with increased or decreased loneliness. Instead, we focused on both the positive and negative effects of ASMU, revealing potential mechanisms for the “double-edged sword” effect of ASMU on loneliness. This study found that ASMU reinforced both interpersonal satisfaction and state-FoMO and indirectly had different effects on trait-FoMO, thus resulting in different paths of effects on loneliness. Positive pathway: ASMU → Interpersonal satisfaction → Loneliness, ASMU → Interpersonal satisfaction → Trait-FoMO → Loneliness; and negative pathway: ASMU → Interpersonal satisfaction → Trait-FoMO → Loneliness. These findings deepen previous research on the relationship between social media use and FoMO and loneliness. Second, the present study somewhat supports media effects theory, which suggests that media use behavior is important in triggering and increasing media-related cognitions. We found that ASMU positively predicted state-FoMO. Finally, the findings suggest that ASMU also has a “double-edged sword” effect on the trait-FoMO, thus deepening our understanding of the connection between social media and FoMO. Overall, this research point to a dialectical view of ASMU's positive and negative effects, emphasizing the mechanisms that underpin them.

This study provides empirical support and valuable insights for weakening the harmful effects of social media use and maintaining the mental health of college students in the mobile internet era. ASMU could strengthen interpersonal satisfaction and thus reduce the trait-FoMO and loneliness among college students. Therefore, college students can maintain good interpersonal relationships and alleviate negative emotions, such as loneliness, through ASMU. However, ASMU can also increase the state-FoMO. Therefore, some matters must be given attention when participating in social media interaction and communication, such as not replying immediately as often and reducing the frequency of checking social media. If the emergency is genuine, college students can be contacted by calling. Moreover, social media companies are urged to design features that help reduce the state-FoMO, such as the FoMO-R method developed by Alutaybi et al. (2020).

### 4.3. Limitations and future research directions

This study also has several limitations. First, although this cross-sectional study validates the proposed model, it still needs stronger evidence to be considered for future longitudinal studies to explore the causal relationships between variables. Second, the present study explored the “double-edged” pathways of ASMU on loneliness from the aspect of “how it affects,” but it is unclear under which conditions these pathways are greater or lesser. Future research needs to consider moderating variables to explore individual differences in the effectiveness of ASMU behaviors. Third, this study did not distinguish between the active use of different social media platforms. Future research could compare whether the explanatory effects of our model differ by the social media platform. Finally, our findings need to be cautiously generalized because our sample only comprises college students. Future research could validate the findings with other social media user groups.

## 5. Conclusion

After controlling for passive social media use (PSMU) and daily SM use time, ASMU was positively associated with interpersonal satisfaction and state-FoMO. In turn, increased interpersonal satisfaction could reduce loneliness directly and *via* lowering the levels of trait-FoMO. However, higher state-FoMO level was associated with increased trait-FoMO, which increased the risk of loneliness. The results suggest a dialectical view of the relationship between ASMU and its psychological consequences and provide a more comprehensive understanding of the relationship between social media use and loneliness.

## Data availability statement

The raw data supporting the conclusions of this article will be made available by the authors, without undue reservation.

## Ethics statement

Ethical review and approval was not required for the study on human participants in accordance with the local legislation and institutional requirements. Written informed consent for participation was not required for this study in accordance with the national legislation and the institutional requirements.

## Author contributions

JM conceived the idea, conducted data analyses, and drafted the manuscript. G-xF conceived the idea and revised the manuscript. J-jH provided advice on some details of this manuscript. All authors contributed to the article and approved the submitted version.
